# Feasibility of portal dosimetry for flattening filter‐free radiotherapy

**DOI:** 10.1120/jacmp.v17i1.5686

**Published:** 2016-01-08

**Authors:** Robert W. Chuter, Philip A. Rixham, Steve J. Weston, Vivian P. Cosgrove

**Affiliations:** ^1^ Leeds Cancer Centre, St James's University Hospital Leeds UK

**Keywords:** electronic portal imaging device, flattening filter free, portal dosimetry

## Abstract

The feasibility of using portal dosimetry (PD) to verify 6 MV flattening filter‐free (FFF) IMRT treatments was investigated. An Elekta Synergy linear accelerator with an Agility collimator capable of delivering FFF beams and a standard iViewGT amorphous silicon (aSi) EPID panel (RID 1640 AL5P) at a fixed SSD of 160 cm were used. Dose rates for FFF beams are up to four times higher than for conventional flattened beams, meaning images taken at maximum FFF dose rate can saturate the EPID. A dose rate of 800 MU/min was found not to saturate the EPID for open fields. This dose rate was subsequently used to characterize the EPID for FFF portal dosimetry. A range of open and phantom fields were measured with both an ion chamber and the EPID, to allow comparison between the two. The measured data were then used to create a model within The Nederlands Kanker Instituut's (NKI's) portal dosimetry software. The model was verified using simple square fields with a range of field sizes and phantom thicknesses. These were compared to calculations performed with the Monaco treatment planning system (TPS) and isocentric ion chamber measurements. It was found that the results for the FFF verification were similar to those for flattened beams with testing on square fields, indicating a difference in dose between the TPS and portal dosimetry of approximately 1%. Two FFF IMRT plans (prostate and lung SABR) were delivered to a homogeneous phantom and showed an overall dose difference at isocenter of ∼0.5% and good agreement between the TPS and PD dose distributions. The feasibility of using the NKI software without any modifications for high‐dose‐rate FFF beams and using a standard EPID detector has been investigated and some initial limitations highlighted.

PACS number: 87.55.Qr

## I. INTRODUCTION

Linear accelerators have traditionally used a conically‐shaped flattening filter to remove the forward peaked profile of the photon beam produced by the target to make treatment planning more manageable for conventional radiotherapy applications.^(1)^ Recent developments of multileaf collimators (MLCs) and more powerful computer planning has allowed flattening filter‐free (FFF) photon beams to be intensity modulated and for complex dose distributions to be created.^2^, ^3^ The advent of beam modulation negates the need for a flattening filter as, in principle, any desired fluence can be created from a combination of many complex shaped segments.^(4)^ Removing the flattening filter increases the dose rate which, in turn, shortens treatment times,^(5)^ reduces head scatter, and equalizes the energy spectra of the photons across the beam.^(4)^ These help to improve patient compliance with treatment, reduce the risk of secondary cancers, and improve the ability of the treatment planning system (TPS) to accurately calculate the dose.^(4)^

Due to the complex nature of intensity‐modulated radiation therapy (IMRT) and volumetric‐modulated arc therapy (VMAT) treatments, the dose to the patient is generally verified by an independent measurement which is usually performed prior to treatment. Two recent documents produced in the UK, *Towards Safer Radiotherapy* ^(6)^ and The National Cancer Peer Review's *Radiotherapy Measures*,^(7)^ have highlighted the importance of *in vivo* dosimetry in verifying complex treatments.

Most modern linacs have an electronic portal imaging device (EPID) attached to the gantry for geometric verification of patient and field position.^(8)^ These EPIDs can also be used for dosimetric verification, either in a pretreatment or *in vivo* context. Early work involved using the central pixels of the EPID to measure the transmitted signal to determine the dose to the isocenter.^(9)^ More recent work has enabled the photon fluence exiting the patient to be imaged and, from this, the dose inside the patient can be reconstructed.^10^, ^11^ *In vivo* portal dosimetry is of particular interest, as this can be used to calculate the 3D dose distribution within the patient based on images acquired during the treatment.^(12)^ The validity of EPID *in vivo* verifications was also illustrated by Mans et al.^(13)^ who found that, out of over 4,300 patient verifications that they had completed between 2005 and 2009, 17 serious errors had been avoided and nine of these would not have been identified by pretreatment verification only. Another group showed that after three years of using the method originally described by Piermattei et al.,^(9)^ ∼10% of plans were out of tolerance, with 1.5% requiring intervention.^(14)^

There are two basic approaches to portal dosimetry: a forward approach and a backward approach. In the first, the measured portal image is compared to a predicted dose/photon fluence at the level of the EPID calculated using a TPS or independent algorithm.^(15)^ The second method uses portal images to reconstruct the dose within the patient using a back‐projection algorithm.^(9)^ This allows the direct comparison of the delivered dose with the intended dose, as calculated using the TPS.^16^, ^17^ The latter method is the most commonly used as it allows 3D reconstruction of the dose, and is the method that is used in this study.^(18)^ The back‐projection method developed at NKI reconstructs the dose within the patient or phantom using a dose‐response matrix ($S_{\text{ij}}$), scatter corrections, inverse square law factor, and transmission factor of the phantom. The $S_{\text{ij}}$ matrix is a measure of the relative dose response of the EPID pixels over its entire surface, determined using an ion chamber. Further ion chamber measurements help to determine scatter correction kernels to account for scatter within the EPID itself, scatter within the phantom, and scatter from the phantom to the EPID.^(19)^ Monte Carlo techniques have also been used by other groups to determine similar scatter kernels; however, this is computationally intensive and can be hard to relate back to the patient.^(20)^ For this study, the transmission is used to correct for attenuation along the beam axis. The factors and kernels combine to form a model that converts portal images to dose. These are applied after the transmission through the phantom, calculated by dividing the portal image by the ‘open image’ — an ‘open image’ being an image of the field delivered directly to the EPID without a phantom or scattering medium in place.

Previous work by Tyner et al.^(21)^ has shown that portal dosimetry can be performed with FFF beams. They found that the panel saturated at 1000 MU/min for field sizes greater than 15×15 cm and used 1 cm of water‐equivalent plastic placed on the EPID surface to counter this. This enabled them to obtain correction factors to determine the absolute dose at the EPID plane. The current study utilizes an alternative method for characterizing the response of the EPID, proposed by the group at the NKI.^(17)^ This method helps to improve the characterization of the EPID and thus increases the accuracy of the dose calculations. This study also uses a linac with an Agility head comprised of 160 MLC leaves, giving higher resolution beam shaping as well as improved accuracy of the leaf positioning and reduced interleaf leakage.^(22)^

This work was initiated to examine the feasibility of using the existing NKI software and an existing EPID detector, both without modification, to verify high‐dose‐rate FFF beams. This is an initial evaluation to determine whether the unmodified software and hardware could provide an immediate and accessible solution for *in vivo* dosimetry to detect gross errors in advanced radiotherapy delivery techniques.

## II. MATERIALS AND METHODS

### A. Characterizing the EPID

A Synergy linear accelerator with an Agility head (Elekta, AB, Stockholm, Sweden) was used in 6 MV FFF mode to characterize the iViewGT (Elekta, AB) (v3.4) a‐Si EPID (RID 1640 AL5P). The FFF beam characteristics for this type of linac have been previously described.^(23)^ Prior to the characterization of the panel, the saturation of the imager was investigated. A series of portal images were acquired by setting a 20×20 cm aperture and delivering 100 MU at a range of dose rates from the maximum dose rate (∼1200 MU/min) to 600 MU/min. The EPID was at a fixed position (160 cm) from the X‐ray source. Image frames were acquired ‘continuously’ (i.e., in an integrated mode) during the irradiation at a frame rate of 1 per∼1.8 MU. The final image was the average of all the image frames acquired during the fields or segment. The resulting images were analyzed by plotting profiles across the images to determine the maximum dose rate at which the images did not saturate.

The characterization was performed using the method developed by NKI^(17)^ which is summarized in Table 1. Absolute dosimetry was performed in a 30 cm×30 cm water‐equivalent (WTe) phantom of thickness 20 cm using a Semiflex chamber (type 31010, PTW GmbH, Freiburg, Germany) calibrated at 10 cm depth with a Farmer‐type chamber (type 30010, PTW GmbH) to enable traceable calibration back to absolute dose. For the field series measurements, nine field sizes were measured between 3 cm×3 cm and 23 cm×23 cm and for the phantom thickness series, 11 thicknesses were measured between 4 cm and 44 cm. The $S_{\text{ij}}$ matrix^(24)^ was measured for a 6 MV flattened beam, as this removes the effect of the flood field, to effectively get back to an ‘uncorrected’ image. All of these measurements form the basis of the model to represent the response of the imager so that it can be used for dose calculation. The characterization measurements were fed into software provided by the NKI^(17)^ which produces the model as described in the Materials & Methods section A above.

Part of the calibration process includes the calculation of a mean offset factor, determined from the differences between the EPID measurements and absolute chamber measurements at the reference field size (10×10 cm) and depth (10 cm).

**Table 1:** Measurements taken to characterize the EPID and used to input into software to create the model.

*Measurement*	*Comment*	*Equipment*	*Phantom* (cm×cm×cm)	*Field Size (cm^2^)*
1. Calibrate ion chamber	For absolute dosimetry and to account for daily output variation	Farmer chamber used to calibrate Semiflex back to absolute dose	30×30×20 slabs	10×10
2. $S_{\text{ij}}$ matrix (and offset)	To measure dose response over entire EPID.	a) Semiflex with a brass buildup in an empty PTW MP3 water tank b) Take 2 EPID images	–	26×26
3. Open‐field series	At 160 cm SSD	a) Semiflex with brass buildup b) EPID	–	Series
4. Phantom field series	Constant phantom thickness, varying field size	a) Semiflex at isocenter of phantom b) EPID	30×30×20 slabs	Series
5. Phantom thickness series	Constant field size, varying phantom thickness	a) Semiflex at isocenter of phantom b) EPID	Series	10×10

### B. Testing the model

The model was validated using three square field sizes delivered to three thicknesses of phantom material. Field sizes of 4×4 cm, 10×10 cm, and 20×20 cm were created on the Monaco v3.3 (Elekta, AB) treatment planning system (TPS) using a 6 MV FFF beam model. These were delivered to 12 cm, 24 cm, and 36 cm thicknesses of 30×30 cm slabs of water‐equivalent (WTe) phantom material. EPID images were captured for portal dosimetry, using standard Elekta imaging parameters. The dose at the isocenter was also measured with an ion chamber to be used as the benchmark. The output of the machine was also measured to account for variation in the linac on a day‐to‐day basis.

The planned dose was then compared to the measured dose using the NKI software.^(17)^ This imports the DICOM CT data files and associated DICOM RT structure and dose objects and uses the model created in the earlier stage (see Materials & Methods section A) to calculate the dose from the images taken during the treatment delivery. The planned and measured dose distributions were compared, using the percentage difference between the planned and calculated isocenter doses.

### C. Verifying treatment plans

**Figure 1:**
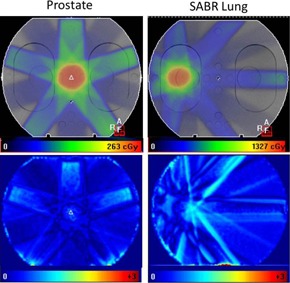
The planned dose distribution from the TPS (top row) for a prostate and SABR lung plan. A gamma analysis using 3%/3 mm is shown in the bottom row comparing the measured dose to the planned dose for each site.

As an initial step towards investigating the feasibility of using software in more realistic, clinical situations, two complex, clinical 6 MV FFF IMRT patient plans were used to test the model. These were plans that had already undergone preclinical validation with Delta^4^ (ScandiDos, Uppsala, Sweden) measurements. Therefore, the plans had all met minimum criteria of 95% 3%/3 mm gamma pass with this system. A 37 Gy in 15‐fraction radical prostate plan (used as a boost to an HDR treatment) and a 55 Gy in 5‐fraction SABR lung plan were used. Both plans were inverse‐planned, with the prostate plan using five equally spaced beams with an average of six segments per beam. The SABR lung plan was composed of seven equally spaced beams with an average of 15 segments per beam. Both these treatments were generated on Monaco,^(25)^ then recalculated on an in‐house IMRT homogeneous phantom as the study set. These were delivered to the phantom with a maximum dose rate of 800 MU/min and the portal images recorded along with open images. The planned and measured dose distributions were compared using a 2D gamma analysis.^(26)^ The dose distribution within the phantom for both plans is shown in Fig. 1.

## III. RESULTS

### A. Characterizing the EPID

**Figure 2:**
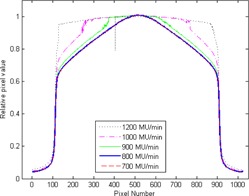
Profiles across the center of the EPID image showing only five different dose rates for clarity. The pixel size is 0.25 mm at isocenter. The high dose rates exhibit saturated profiles which become less saturated until, at 800 MU/min, the images are no longer saturated. Note that the 800 MU/min (thick blue line) and 700 MU/min (dashed red) lines are superimposed.

A subset of the profile measurements captured on the EPID as the dose rate was reduced from the maximum of ∼1200 MU/min to 600 MU/min in eight steps, delivering a 100 MU 20 cm×20 cm field are shown in Fig. 2. A dose rate of 800 MU/min was found to be the threshold above which panel saturation occurred.

**Figure 3:**
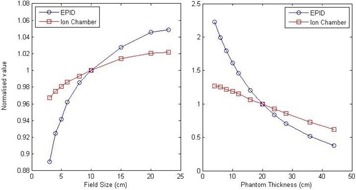
Examples of the characterization measurements showing the open‐field size series (left) and phantom thickness series (right). The plots show the sum of the central 16×16 pixels of the EPID images (blue circles) and the ion chamber measurements (red squares). The pixel size is 0.25 mm at isocenter.

The panel was characterized as described in the Materials & Methods section above, taking several series of measurements with both an ion chamber and with the EPID. Two of these series can be seen in Fig. 3, which shows the ion chamber data and central 16×16 square pixels of the EPID images. These data are shown for the field‐size series and the phantom thickness series.

For the FFF EPID characterization, the mean offset correction factor was measured to be 2.4%. This compares with a factor of 2.7% measured for the flattened beam EPID characterization.

### B. Testing the model

The resulting dataset was analyzed using the NKI software^(17)^ which fits the data in several stages to determine the response of the EPID. Once the model was created, simple square‐field plans were generated in Monaco to test the model was performing correctly. The results for fields of three sizes delivered to three thicknesses of phantom material are shown in Table 2. For comparison, flattened beam data are also shown using identical field sizes and phantom thicknesses delivered on the same Agility head. The dose difference (Δ_dose_) between the planned and measured isocenter doses are shown as a percentage. The difference between the measured dose and planned dose were within ± 1% for most field sizes and phantom thicknesses, which indicated the model was accurately calculating the dose to the isocenter and the dose distribution across the field. The 20 cm×20 cm field delivered to a 36 cm phantom differed by more than 5% from this value. Chamber measurements taken within the fields were consistent with the planned values.

### C. Verifying plans

The two FFF IMRT treatments created on Monaco were delivered to the homogeneous IMRT phantom and EPID images acquired. The images were analyzed with the validated model and the results can be seen in Table 3 and Table 4, for the prostate and lung plans, respectively. These illustrate that with the corrected model the differences in calculated and planned doses are generally low, with the total dose difference well within acceptable clinical criteria. The gamma analyses for these treatments also indicate that the planned and measured dose distributions are generally in good agreement (see Fig. 1), achieving clinically acceptable gamma values (>95% of points within 3%/3 mm). This tolerance value was breached in four of the seven beams in the second, SABR plan, with 93.5% of points passing the total plan. The most notable difference was for beam 1, which had a dose difference of 8.7% and 87.6% of points achieving 3%/3 mm. There is no explanation to account for this difference. However, the Delta^4^ measurements (see 3, 4), by contrast, are well within tolerance for this and all individual beams for the plan. If a less stringent gamma criteria of 5%/3 mm is used (also included in 3, 4), both the prostate and lung SABR plans would pass overall.

**Table 2:** The difference in dose between the planned and measured doses are shown for different field sizes and phantom thicknesses. The dose differences (Δ_dose_ (%)) are show for flattened and FFF beams.

	4 cm×4 cm		10 cm×10 cm		20 cm×20 cm	
*Field Size*	*6 MV*	*6 MV FFF*	*6 MV*	*6 MV FFF*	*6 MV*	*6 MV FFF*
12 cm	‐3.5	+0.2	+0.1	‐0.1	+1.0	+2.1
24 cm	‐0.4	+0.8	+0.1	+0.1	‐0.6	‐0.6
36 cm	‐2.5	+0.9	‐0.3	+0.4	‐3.1	‐5.2

**Table 3:** IMRT FFF prostate results showing the difference in dose between the planned and measured doses for the different fields. Also shown are the percentage of points with a gamma value less than or equal to 1, using a global gamma of 3%/3 mm and 5%/3 mm. Measurements taken with a Delta^4^ are also included.

	*ANT*	*LAO*	*LPO*	*RPO*	*RAO*	*Total*
$\Delta_{\text{dose}}(\%)$	‐0.3	‐1.6	+3.1	+0.9	+0.4	+0.5
$\gamma \leq 1(\%)\;\text{at}\;3\%/3\;\text{mm}\;{(\text{Delta}}^{4})$	100	100	99.3	100	100	100
γ≤1(%) at 3%/3% mm	100	100	99.6	99.0	100	98.7
γ≤1(%) at 5%/3% mm	100	100	100	100	100	99.9

**Table 4:** IMRT FFF SABR lung results showing the difference in dose between the planned and measured doses for the different fields. Also shown are the percentage of points with a gamma value less than or equal to 1, using a global gamma of 3%/3 mm and 5%/3 mm. Measurements taken with a Delta^4^ are also included.

	*1 (145°)*	*2 (196°)*	*3 (247°)*	*4 (298°)*	*5 (349°)*	*6 (40°)*	*7 (91°)*	*Total*
$\Delta_{\text{dose}}(\%)$	‐8.7	+2.1	+2.8	‐1.8	‐2.0	+1.2	+0.4	‐0.6
$\gamma \leq 1(\%)\;\text{at}\;3\%/3\;\text{mm}\;{(\text{Delta}}^{4})$	100	100	100	100	100	100	100	100
γ≤1(%) at 3%/3 mm	87.6	97.8	100.0	100.0	89.3	87.0	92.7	89.5
γ≤1(%) at 5%/3 mm	89.8	98.0	100	100	90.3	88.8	94.7	99.3

## IV. DISCUSSION

A dose rate of 800 MU/min allows the EPID (RID 1640 AL5P) to be characterized, but this dose rate is lower than that which would be used clinically for FFF beams. However a dose rate of 800 MU/min is higher than the dose rate used for flattened beams and demonstrates the feasibility of using the software and hardware described here for FFF beam verification without any specific modification. Future developments in the design of the panel and image acquisition should mean that images can be acquired at even higher dose rates using the methods described here.

The results for the two complex IMRT treatment plans delivered to a homogeneous phantom demonstrates that there is about ± 0.5% difference between the planned and measured doses at isocenter using this method. The agreement between the planned and measured dose distributions indicate the potential the software could have in validating clinical plans known to be acceptable in terms of dose and dose distributions. The results also indicate the feasibility of FFF portal dosimetry using the current software without the need for modification to account for the FFF beam energy spectrum, beam properties, and dose rate. However, from the limited plan data used, the indications are it is likely that broader tolerances would need to be used so that false‐positives would not make use of the system too inefficient. As portal dosimetry is expected to be used to detect gross errors, rather than be an accurate plan verification tool, a gamma criteria of 5%/3 mm would be appropriate, as observed previously by other investigators.^27^, ^28^

This work in principle would indicate that the portal dosimetry approach should be capable of detecting gross errors (≥10%) ^(29)^ in the treatment pathway. The ability of portal dosimetry to catch errors such as delivering incorrect IMRT segments has been shown by Mans et al.,^(13)^ illustrating that it should be sensitive enough to catch dosimetric errors of ∼6% or less. However this would need to be further tested in more challenging, realistic clinical situations — for example, in an inhomogeneous phantom/patient with substantially more case studies — to understand the statistical variation and uncertainties.

Planning systems, such as Monaco, used in this study have an inherent uncertainty of ∼1% due to the variance set in the Monte Carlo calculation of the TPS. This sets the lowest possible dosimetric error that can be detected. However, the linac has tolerances associated with radiation delivery, meaning that, for example, an MLC leaf can be up to half a millimeter from its expected position or the output of the linac could be a few percent different from the nominal value but still be within tolerance. The setup of the patient and anatomical changes within the patient also generate uncertainties^(30)^ and, although image‐guided radiotherapy (IGRT) can assist in limiting systematic and random uncertainties, some setup errors may still be present. The process of determining the delivered dose using the method discussed here again adds to the uncertainty. This means that, in general, the identification of dosimetric errors much less than 5% is difficult to achieve without producing a significant number of false‐positives. The measurements upon which the model is based provide one source of error and may be the most significant. The model uses these measurements to derive the scatter kernels in order to correct for scatter within the EPID, between the phantom and the EPID, and within the phantom itself. It derives these by iteratively fitting the EPID data to the ion chamber measurements.

It has been demonstrated here that the verification of IMRT treatments is possible with this method, but with some limitations. In principle the technique could be transferable to VMAT treatments^(31)^ but they were not investigated here due to current issues with data logging the gantry angle whilst using iViewGT. The software deals with IMRT and VMAT in a similar way so could in principle handle both techniques.

Even though this model calculates doses to within 1% of the planned doses for most fields, there remain larger differences in the isocenter doses (∼5%) for the 20×20 cm field size delivered to 36 cm of WTe. It is thought that this could be because dose‐rate effects and scatter kernel differences between flattened and nonflattened beams would have a greater effect on larger fields and deeper regions. However, this would need to be investigated more comprehensively.

One inconvenience with the method used here is the need to take open images for each field that is delivered. Other work has been done to remove the need for these fields in the future. Pecharromán‐Gallego et al.^(32)^ have created a modification to the back‐projection method to calculate the transmission through the patient/phantom without the need for open images. This would speed up the process in the future and may allow FFF portal dosimetry to be performed with dose rates greater than 800 MU/min, as there will no longer be a problem of saturation since the material in the beam lowers the dose rate and no open images are needed. This is not currently used because the method is less accurate than using open images and has not been verified clinically.

## V. CONCLUSIONS

*In vivo* dosimetry can help to ensure safe and effective radiotherapy. Portal dosimetry utilizing electronic portal imaging devices (EPID) is an existing technique that has been shown to work well with standard, flattened radiotherapy beams. With the emerging use of high‐dose‐rate flattening filter‐free (FFF) radiotherapy there is an associated need to verify these treatments efficiently. This study has shown that standard, iView GT EPID (RID 1640 AL5P) saturates when exposed to a dose rate greater than 800 MU/min. However 800 MU/min can be used to characterize the EPID response in a 6 MV FFF beam and create a model to calculate the portal dose, using software developed at the NKI, without the need for modification of the software for FFF beams. The model was tested using a small sample of square fields of different sizes delivered to a range of phantom thicknesses. It was found the model does not accurately predict doses for larger fields (20 cm×20 cm). Identical measurements were taken with a model created with flattened data and showed that the dose differences are similar for both FFF and flattened beams.

The model was also tested for feasibility with two IMRT treatments delivered to a homogeneous phantom. This only covered two treatment types, both of which have relatively small PTVs with volumes of about 65 cm^3^. Further work is required to investigate how well this method will work in more realistic clinical situations, for example in an inhomogeneous phantom/patient and with more patient studies, to understand statistical variation and uncertainties. In addition it will be important to investigate treatment plans, such as head and neck IMRT, which generally use larger treatment field segments. However the feasibility of using the NKI software unmodified with FFF beams has been investigated and some initial limitations highlighted.

## ACKNOWLEDGMENTS

The authors would like to thank NKI‐AVL and Elekta for allowing us to use their portal dosimetry software for this study, with a special thank you to Igor Olaciregui‐Ruiz. This software forms part of an Elekta research project aimed at developing a commercial portal dosimetry product. Finally, I am grateful to the referees for helping to improve the paper significantly.

## Supporting information

Supplementary Material
