# Supplementary figures and images for: Feasibility of portal dosimetry for flattening filter‐free radiotherapy
Source: J Appl Clin Med Phys. 2016 Jan 8;17(1):112–20. doi: 10.1120/jacmp.v17i1.5686 (PMC5690198; doi:10.1120/jacmp.v17i1.5686)

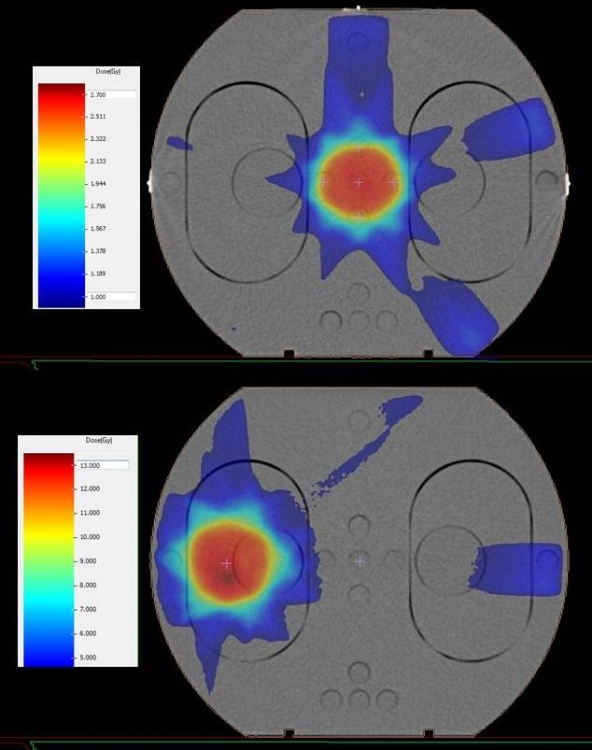

Supplement: Supplementary file 1 — Supplementary Material [file ACM2-17-112-s001.jpg]
